# Increased Expression of Matrix Extracellular Phosphoglycoprotein (MEPE) in Cortical Bone of the Rat Tibia after Mechanical Loading: Identification by Oligonucleotide Microarray

**DOI:** 10.1371/journal.pone.0079672

**Published:** 2013-11-08

**Authors:** Christianne M. A. Reijnders, Huib W. van Essen, Birgitte T. T. M. van Rens, Johannes H. G. M. van Beek, Bauke Ylstra, Marinus A. Blankenstein, Paul Lips, Nathalie Bravenboer

**Affiliations:** 1 Department of Internal Medicine, Endocrine Section, VU University Medical Center, Amsterdam, The Netherlands; 2 Department of Clinical Chemistry, VU University Medical Center, Amsterdam, The Netherlands; 3 Faculty of Human Movement Sciences, VU University, Amsterdam, The Netherlands; 4 Department of Clinical Epidemiology and Biostatistics, VU University Medical Center, Amsterdam, The Netherlands; 5 Department of Pathology, VU University Medical Center, Amsterdam, The Netherlands; 6 Research Institute MOVE, Amsterdam, The Netherlands; INSERM U1059/LBTO, Université Jean Monnet, France

## Abstract

Skeletal integrity in humans and animals is maintained by daily mechanical loading. It has been widely accepted that osteocytes function as mechanosensors. Many biochemical signaling molecules are involved in the response of osteocytes to mechanical stimulation. The aim of this study was to identify genes involved in the translation of mechanical stimuli into bone formation. The four-point bending model was used to induce a single period of mechanical loading on the right tibia, while the contra lateral left tibia served as control. Six hours after loading, the effects of mechanical loading on gene-expression were determined with microarray analysis. Protein expression of differentially regulated genes was evaluated with immunohistochemistry. Nine genes were found to exhibit a significant differential gene expression in LOAD compared to control. *MEPE*, *Garnl1*, *V2R2B*, and *QFG-TN1 olfactory receptor* were up-regulated, and *creatine kinase* (*muscle form*), *fibrinogen-B beta-polypeptide*, *monoamine oxidase A*, *troponin-C* and *kinesin light chain-C* were down-regulated. Validation with real-time RT-PCR analysis confirmed the up-regulation of *MEPE* and the down-regulation of *creatine kinase* (*muscle form*) and *troponin-C* in the loaded tibia. Immunohistochemistry showed that the increase of MEPE protein expression was already detectable six hours after mechanical loading. In conclusion, these genes probably play a role during translation of mechanical stimuli six hours after mechanical loading. The modulation of MEPE expression may indicate a connection between bone mineralization and bone formation after mechanical stimulation.

## Introduction

The ability of the skeleton to form bone after mechanical stimulation is a very complicated process, which consists of mechanosensing, mechanotransduction and the bone formation response. It is well-known that osteocytes function as mechanosensors via canalicular processes and communicating gap junctions in the early stage of bone remodeling [1,2]. Many biochemical signal molecules are involved during mechanical transduction, finally, resulting in bone formation.

*In vivo* studies in animals report glucose-6-phosphate dehydrogenase (G6PD)[3,4], c-fos [5,6], cAMP [7], COX‑2 [8], NO and prostanoid [9], insulin-like growth factors (IGFs)[6,10,11], transforming growth factor-β (TGF-β)[6,10], protein kinase B (PKB or Akt)[12] , glutamate transporter (GLAST)[12], wnt/β-catenin [13,14] and sclerostin [14,15] as biochemical signal molecules during the translation of mechanical loading into bone formation. The responses to *in vivo* mechanical loading are time-dependent. Early strain related changes within 5 minutes after loading are shown in osteocytes in which the glucose-6-phosphate dehydrogenase (G6PD) activity is increased [3,4]. The cAMP level is also rapidly increased after loading [2,7]. COX‑2 [8], nitric oxide (NO), and prostanoid [9] are also important for bone formation elicited by mechanical strain. These early events are followed by changes in gene expression level. Compression of caudal vertebrae results in enhanced c-fos mRNA expression in cortical and trabecular osteocytes 30 minutes after loading [5]. Four-point-bending of the tibia shows an up-regulation of c-fos mRNA expression in the periosteum within 2 hours after loading and an increase of IGF‑I and TGF-β mRNA in the periosteum within 4 hours after loading [6]. Increased IGF‑I mRNA expression is observed on trabecular surfaces and in osteocytes of the diaphysial cortex (cortical and trabecular osteocytes) of rat caudal vertebrae within 6 hours after a single loading session, followed by an increased expression of type I collagen and osteocalcin on the bone surface [11]. In addition, the concentrations of IGF‑I protein within the humerus increased after 6 weeks of weightbearing exercise (5 days/week) in the rat-with-backpack model, whereas the concentrations of TGF-β protein decreased [10].

 All of the above mentioned studies observed one single or a few mediators of the osteogenic response, but the microarray technology, used in this study, allows exploring multiple genes at once. The aim of this study was to identify candidate genes that are involved in the translation of mechanical stimuli into bone formation. Load was applied using four-point bending. This model was chosen because adequate bone adaptation was demonstrated previously [16,18,26]. The results from the microarray experiment were confirmed both at RNA level by RT-PCR and at protein level by immunohistochemistry (IHC). 

## Materials and Methods

### In vivo mechanical loading experiments

Three separate mechanical loading experiments were performed to obtain tibia material suitable for detection of RNA expression in microarray and for detection of protein expression by immunohistochemistry, (table 1). Mechanical loading was applied using the 4-point bending system of Forwood and Turner [16,17] and was performed under general anesthesia (2% isoflurane in 1 l/min O_2_ and 2 l/min N_2_O). The animal experiments were in accordance with the governmental guidelines for care and use of laboratory animals and approved by the Institutional Animal Care and Use Committee (IACUC) of the VU University Medical Center Amsterdam, the Netherlands. The animals were kept under standard laboratory conditions.

**Table 1:** SET UP OF THREE DIFFERENT LOADING EXPERIMENTS.

Experiment	Rationale	N	Group	Experimental condition of:		Endpoints
				Right tibia	Left tibia		
A	Explore differential expression of genes due to bending, not squeezing	9	LOAD	Single bending load	contra lateral control	RNA isolation for micro array analysis and RT-PCR	
		10	SHAM	Sham load	contra lateral control		
B	Confirmation of micro array differences at protein level	5	LOAD	Single bending load	contra lateral control	Histology and IHC	
		5	CONTROL	control	contra lateral control		
C	Differential expression due to repeated bending.	9	REPEATED LOAD	repeated bending load	contra lateral control	Histology and IHC	

Rationale and outline of the experimental setup of the three different experiments(exp) performed.. In experiments A and B a single bout of loading was applied for both RNA analysis (experiment A) as well as protein localization by immunohistochemistry (IHC) (experiment B). In experiment C repeated loading was applied for studying cumulative protein expression.

In experiment A nineteen female 12-week-old Wistar rats (Harlan, Zeist, The Netherlands) were randomly assigned to two weight-matched groups: LOAD (223 ± 14 g, n = 9) and SHAM (228 ± 10 g, n = 10). The right tibiae of rats in the LOAD group underwent “medio-lateral” loading (distance between the centers of the loading pads: upper pads: 11 mm and lower pads: 23 mm). The left tibia served as contra lateral control to adjust for systemic effects of the bending. The right tibia of rats in the SHAM group underwent sham loading (opposed pads were placed at the inner position: 11 mm). The left tibia served as contra lateral control to adjust for systemic effects of the squeezing (sham-loading). Since loading will result in bending and squeezing of the tibia and sham-loading only in squeezing of the tibia; the SHAM group was used as control for the LOAD group. A single loading episode constituted 300 cycles (2 Hz) using a peak magnitude setting of 60 N as described before [18]. Measurement with an external force reader before and after bending revealed that the actually used force was 72 ± 3 N (mean ± SD). All left tibia served as contra lateral control. The rats were sacrificed by CO_2_ asphyxiation exactly six hours after loading. This time-point was based on a previous study of Lean and colleagues [11] and on our own *in situ* hybridization studies [18,19]. The tibiae were dissected and after removal of the proximal and distal end, periosteum and bone marrow, immediately frozen in liquid nitrogen and stored at -80 °C until RNA isolation.

In experiment B ten female 12-week-old Wistar rats (Harlan, Zeist, The Netherlands) weighing 235 ± 12 g were randomly assigned to two groups: LOAD (n=5) and CONTROL (n=5). The right tibiae of rats in the LOAD group underwent “medio-lateral” loading identical to the rats in the LOAD group of experiment A, while the left tibiae of these rats served as a non-loaded contra lateral control. The rats in the CONTROL group did not receive any mechanical loading. All rats were sacrificed by CO2 asphyxiation six hours after loading. The tibiae were dissected and the proximal condyles were sawn off. The tibiae were then fixated in 4% (w/v) paraformaldehyde buffered in PBS at 4 °C for 24 hours and decalcified in 10% EDTA with 0.5% paraformaldehyde in PBS at 4 °C for 30 days. The tibiae were dehydrated with ethanol and xylene and embedded in paraffin.

In experiment C nine female 12-week-old Wistar rats (Harlan, Zeist, The Netherlands) weighing 234 ± 12 g were assigned to the REPEATED LOAD group. The right tibia of these rats underwent for two weeks, five days a week, a single episode of “medio-lateral” loading comprising 300 cycles (2 Hz) using a peak magnitude setting of 40 N. Six hours after the final loading episode the rats were sacrificed by CO2 asphyxiation. The tibiae were dissected, the proximal condyles were sawn off and the tibiae were prepared for histology identically to experiment B.

### RNA isolation

The diaphysis of the tibia from experiment A was pulverized in presence of Trizol (Invitrogen) using the Freezer mill 6750 (Spex Certiprep, Metuchen, NY, USA) subsequently followed by a Trizol extraction, a chloroform/isoamylalcohol extraction, and a second Trizol extraction according to the manufacturer’s instructions. The RNA pellet was dissolved in RNase-free water and stored at -80°C prior to use. Thus the sample included total RNA from the tibia shaft, containing osteocytes, osteoblasts, lining cells and intracortical blood vessel cells. For the real-time RT-PCR analysis the samples were additionally treated with RNase-free DNAse to eliminate DNA contamination.

The quality of the RNA samples was determined with the RNA 6000 Nano Assay Kit (Agilent Technologies). The yield of RNA was measured with the spectrophotometer (A_260_)(NanoDrop® ND‑1000 Spectrophotometer). The mean yield of total RNA samples used for the microarray analysis was 1604 ± 501 ng/μl (mean ± SD) ranging from 18.6 μg to 48.9 μg per tibia with an A_260_/A_280_ ratio of 1.97 ± 0.05 (mean ± SD).

### Microarray, probe generation, hybridization and washing

Custom made microarrays were used [20]. The total number of spotted oligonucleotides was 4803 per region, including 52 spots of the housekeeping gene glyceraldehyde-3-phosphate dehydrogenase (GAPDH).Furthermore, there were 138 empty spots. The slides exhibited a crossreactivity of 10%; every spotted oligonucleotide has a change of 10% that a part of the spotted sequence interacts with a different gene than mentioned in the gene name list.

RNA from five rats of each group was used for microarray analysis cDNA probes were generated from 15 µg total RNA with an oligo-dT ((dT)_20_-VN) primer (Isogen, Maarssen, The Netherlands) and SuperScript™ II Reverse Transcriptase (Invitrogen), with incorporation of aminoallyl-dUTP (Ambion). Probes were indirectly labeled with Fluorolink Cy3 or Cy5 mono-functional dyes according to a dye-swap design. The dye-swap design was used to avoid a possible bias caused by the molecular structure of Cy3 and Cy5. The treated tibia (LOAD or SHAM) was hybridized together with its own contra lateral control tibia creating unique intra-rat hybridization.

The hybridization protocol was adapted from Snijders and colleagues [21] with minor modifications. Pre-hybridization was performed in a hybridization mixture containing 60 µg yeast tRNA (ribonucleic acid transfer, Sigma), 12 µg polyA (Amersham Biosciences) and 24 µg human Cot-1 DNA (Invitrogen) and 30 µg salmon sperm DNA (Invitrogen) in a total volume of 29.2 µl per array. After precipitation the pellet was dissolved in mastermix (50% formamide, 2xSSC, 9.4% dextran sulphate) and 0.2% SDS in a total volume of 130.2 µl per array. Pre-hybridization was maintained for 45 min at 37°C in the Hybridization Station (Genetac^TM^ hybridization station or HybArray12 (Perkin Elmer)). The pre-hybridization mix was gently removed and slides were dried by centrifugation at 200 g for 3 min. The probe-hybridization mixture contained 16.0 µl Cy3 and 16.0 µl Cy5 labeled samples and 26.2 µl hybridization mixture (60 µg yeast tRNA, 12 µg polyA and 24 µg human Cot-1 DNA). After precipitation the pellet was dissolved in mastermix and 0.2% SDS in a total volume of 130.2 µl per array. Probe mix was denaturated at 70°C for 15 min and incubated at 37°C for 60 min. Hybridization was initiated and maintained for 16 h at 30°C in the hybridization station, while the hybridization mixture was agitating. After hybridization, excess hybridization mixture was automatically rinsed off with 50% formamide, 2xSSC, pH 7.0 at 35°C and followed by a wash step with PN buffer (0.1 M sodium phosphate, 0.1% Igepal CA630, pH 8) at 25°C. Excess salt was removed by subsequently rinsing in 0.2xSSC, 0.1xSSC and 0.01xSSC at 25°C. Slides were dried by centrifugation at 200 g for 3 min. 

After drying, the slides were scanned at 10 µm resolution for Cy3 and Cy5 intensities using the microarray scanner (Agilent Technologies) operated by Agilent Scan Control software and Feature Extraction software. Array images were processed with BlueFuse version 3.0 for microarrays (BlueGnome, Cambridge, UK). The dataset is submitted to GEO gene expression omnibus (GSE50707) with platform accession number: GPL: 1397.

### Microarray statistics differential gene-expression

Automatically flagged genes (spots with a confidence value < 0.11) and manually flagged genes (dirty spots and spots with a confidence value between 0.11‑0.15 with a low intensity or bad morphology) were excluded from further analysis. The duplicates of the Cy3 and Cy5 intensities were not averaged, but were treated as separate spots in the analysis. The log_2_ values of the Cy5 to Cy3 ratios were normalized in an intensity dependent fashion (lowess). Genes with more than one missing value across arrays were excluded from the statistical analysis.

Differentially expressed genes were identified by fitting a separate linear model to the expression data for each gene using the language R (http://www.r-project.org). Duplicate spots for each gene, printed on each array, were taken into account [22]. Differential expression was analyzed using moderated t‑statistics based on empirical Bayes estimation [22] and P-values were adjusted according to the linear step-up method of Benjamini and Hochberg to reflect the false discovery rate [23,24]. Genes with a false discovery rate <20% were considered to reflect statistical significance.

Estimated relative differential expression for LOAD (i.e., relative to its contra lateral control) was compared to the estimated relative differential expression for SHAM (i.e. relative to its contra lateral control). Thus, aspects of the experimental intervention bending and squeezing were compared with squeezing resulting in the net effect of bending.

### Microarray statistics single channel analysis

Possible differences between the contra lateral control of the LOAD group and the contra lateral control of SHAM group were investigated. Analyzing the differences between contra lateral control of both groups necessitated single channel analysis to compare the intensities of the control samples between arrays rather than the ratios between the two channels. This analysis was performed on 1944 spots, because missing values could not be handled by the single analysis [25].

### Real-Time RT-PCR

Quantitative RT-PCR analysis was performed on the nine differentially expressed genes to validate the microarray results. RNA from LOAD (n=8) and SHAM (n=10) was used for quantitative RT-PCR analysis. One hundred ng of total RNA was reverse-transcribed using 10 ng/μl random primers (Roche, Basel, Switzerland) and 5 U/μl M-MLV Reverse Transcriptase (Promega) in a mixture containing 5 mM MgCl, 1x RT-buffer, 1 mM dNTPs each, 1M betaine and 0.40 U/μl RNAsin for 10 min at 25°C, 1h at 37°C and 5 min at 95°C in a total volume of 20 μl. All RNA samples were assayed in triplicate. Three μl of cDNA was amplified by PCR using the primers as described in Table 2. For every gene two primer sets were developed. The first primer set amplified a fragment of the mRNA which overlaps with the oligonucleotide that was spotted on the microarray and the second primer set amplified a fragment of the mRNA that was not present on the array. Both primer sets were used, because the microarray had a crossreactivity of 10%.

**Table 2:** PRIMERS FOR REAL-TIME RT-PCR.

			Primer set I						Primer set II				
Accession nr	Target Array (nt)		Forward(5’-3’)	Nucleotide Position (nt)	Reverse (5’-3’)	Nucleotide Position (nt)	Product (bp)		Forward(5’-3’)	Nucleotide Position (nt)	Reverse (5’-3’)	Nucleotide Position (nt)	Product (bp)
AF260922	P1311-1375		Agaacaagccaccctacacg	1214	Cccactggatgatgactcact	1342	129		Aggctgtgtctgttggactg	66	Aagtggatgttgccttggtt	165	100
NM_020083	3625-3689		agagcacggtacctgcagac	3604	tggtagggagctggagagaa	3698	95		tgccttctgaatctgatgactc	3184	agtcaatggaatccccatca	3290	107
NM_012530	1102-1166		cttccgaggtcgaacaggt	1100	tacttctgcgcagggatcat	1214	115		atctggcacaacgacaacaa	718	ggcggaaaacttccttcata	823	106
U05675	1097-1161		accgaggccaacaagtacc	1093	tgtggatggtcatggttctg	1207	115		acggaatactgccacactcc	664	acctcccttccgaatgatct	753	90
U05675	1097-1161		cgaaatggaggactggaaag	1032	gccagcagtccctttgtact	1146	115		tgctacagggtgtgagttgc	324	aggtgaccgaagaggtctca	439	116
AF053990	655-719		ccactgttgacaggagcatc	608	tcacttggagctcacagttct	712	105		cggcttatatccatgcatcc	121	tccttggaggcttcaacatc	226	106
D00688	1460-1524		aaggatgttccagccattga	1441	ccacagaagtggaaacacca	1540	100		accagagcttccacctgaga	858	tcatgcagccacaatagtcc	979	122
J00793	347-411		ggaactggctgagtgtttcc	351	tcctcgtctgtcacatgctc	461	111		gctgcctttgacatgtttga	133	ttcctctttggtgggtgtct	234	102
AF091565	278-342		ctggttcaaacatcccttgc	280	acaggccagtttcaggacag	378	99		cccaggaagaccatctcctt	73	catgctcaggagcacacact	162	90
M75148	2123-2187		catcttgaggacaggcgtta	2089	cagcacatgcctcactccta	2184	96		cagccctaggagcttgtcag	1904	tggcaaagctacatgtatccag	2003	100
PBGD	–		–	–	–	–	–		atgtccggtaacggcggc	1	caaggttttcagcatcgctacca	135	135

Primer set I contained the sequence of the oligonucleotide as spotted on the microarray; Primer set II contained a fragment of the gene which was not present on the array; target array, position oligonucleotide fragment as spotted on the microarray; PBGD, house keeping gene porphobilinogen deaminase;

In each case, the PCR consisted of an initial denaturation step for 3 min at 95°C, followed by 40‑50 cycles (15 sec at 95°C, 1 min at 60°C) in a total volume of 25 μl containing 300nM primers and SYBR Green Supermix (BioRad, Hercules, CA, USA). After the PCR, a melting curve was run from 50°C to 95°C to check the specificity of the reactions.

### Real-Time RT-PCR statistics

All mean Ct (threshold cycle) values were normalized for the house keeping gene PBGD (porphobilinogen deaminase)(2^-δCt^ value; δCt = Ct _GENE OF INTEREST_ – Ct _PGBD_), followed by a natural log-transformation to obtain a normal distribution, which was shown with a Kolmogorov-Smirnov test (SPSS, version 9.0). The paired samples t-test (SPSS, version 9.0) was used to examine differences between the treated right and control left tibia in the same rat. The independent samples t-test (SPSS, version 9.0) was used to compare indirect differences between the LOAD and SHAM group (2^-δδCt^ value; δδCt = δCt _TREATED_ – δCt_control_). A P-value < 0.05 was considered to reflect statistical significance.

### Immunohistochemistry

From both tibiae of the rats from experiment B and C, sections of 5 µm thick were cut. Sections were rehydrated and endogenous peroxidase was quenched with 3% H_2_O_2_ in 40% methanol/PBS. Antigen retrieval was performed by incubation with 1% trypsin for 15 minutes at 37 °C. After blocking of the non-specific binding sites with blocking reagents for 1 hour the sections were incubated overnight at 4°C with 1/200 rabbit-anti MEPE antibody (Santa Cruz sc-68923, CA, USA).The sections were incubated 1 hour with 1/100 biotinylated goat-anti-rabbit antibody (Dako) and 1 hour with horse radish peroxidase labeled streptavidin (Invitrogen). For color development the sections were incubated with AEC reagent (Zymed) and the sections were counterstained with hematoxilin. Quantitative evaluation of the specific staining for MEPE was performed with NIS Elements digital imaging software (Nikon). Digital images were made of the medial cortex of two sections of each tibia at 200x magnification. In the images the area of MEPE positively stained osteocytes was measured and expressed as a percentage of cortical area to obtain the percentage MEPE positive area. Positive staining in capillary blood vessels was excluded from the measurements.

### Immunohistochemistry statistical analysis

Data from two sections of each tibia were averaged. Differences in staining between right and left tibiae were tested with a Wilcoxon matched pairs test. Statistical analysis was performed with Graphpad software. A P-value < 0.05 was considered to reflect statistical significance**.**

## Results

### Identification of differentially expressed mechanosensitive genes with microarray analysis

After severe filtering of spot quality, 2375 spotted genes of the 4803 genes, including 51 housekeeping genes, were retained in the analysis. Comparison of the LOAD tibia and its contra lateral control tibia resulted in nine significant differentially expressed genes with a false discovery rate < 20%. Four genes showed an up-regulation ranging from 1.4-fold to 1.8-fold and five genes were down-regulated (table 3).

**Table 3:** DIFFERENTIALLY EXPRESSED GENES 6 HOURS AFTER MECHANICAL LOADING.

Accession nr	Gene name	M	A	FDR (%)	B	Biological connection
AF260922	matrix extracellular phosphoglycoprotein (MEPE) osteoregulin; osteoblast/osteocyte factor 45 (OF45)	0.87	11.60	5.4	2.35	skeletal development; regulation of bone remodeling; negative regulation of bone mineralization;
NM_020083	GTPase activating RANGAP domain-like 1 (garnl1); tuberin-like protein 1 (tulip 1); GAP-related interacting protein to E12 (GRIPE)[47]	0.51	11.61	5.4	1.92	regulation of transcription
NM_012530	creatine kinase (muscle form) (CKM)	-1.39	9.24	6.5	1.44	phosphocreatine biosynthesis and metabolism
U05675	fibrinogen B beta chain	-1.18	10.18	7.4	1.08	blood coagulation; wound healing; regulation of blood pressure; positive regulation of cell proliferation
AF053990	tissue-type vomeronasal neurons putative pheromone receptor V2R2B	0.68	7.84	9.4	0.56	related to Ca^2+^-sensing receptor and metabotropic glutamate receptors
D00688	monoamine oxidase A	-2.00	8.75	9.4	0.50	behavior; catecholamine catabolism and metabolism; dopamine catabolism; electron transport; neurotransmitter catabolism
J00793	troponin-C	-1.96	7.64	9.4	0.42	Ca^2+^-binding-subunit of the troponin complex
AF091565	QFG-TN1 olfactory receptor	0.55	7.64	14.5	-0.06	G-protein coupled receptor protein signaling pathway; perception of smell, sensory transduction of chemical stimulus
M75148	kinesin light chain C	-1.06	7.88	16.0	-0.25	–

Nine genes were found to exhibit a significant differential gene expression (FDR < 20%) in loaded tibia compared to contra lateral control tibia measured with microarray analysis. This table shows their gene IDs, gene names and their biological connection (PubMed nucleotide; Rat Genome Database).

M ^2^,log (LOAD/control); A positive value of M represents an up-regulation and a negative value of M represents a down-regulation in the loaded tibia compared to the non-loaded contra lateral control tibia; A, average log intensity (^2^log ((LOAD)*(control))^0 5^ ; FDR, false discovery rate; B, posterior odds; B, natural log (chance that a gene is differentially expressed)/(chance that a gene is not differentially expressed);

 Comparison of the SHAM tibia and its contra lateral control tibia did not result in significant differentially expressed genes (data not shown). Furthermore, the nine genes that were differentially expressed between the LOAD and control comparison were not present in the 189 highest ranked genes of the SHAM versus control comparison. This ranking was based on the posterior odds. 

### Validation of differentially expressed genes with real-time RT-PCR

**Figure 1:**
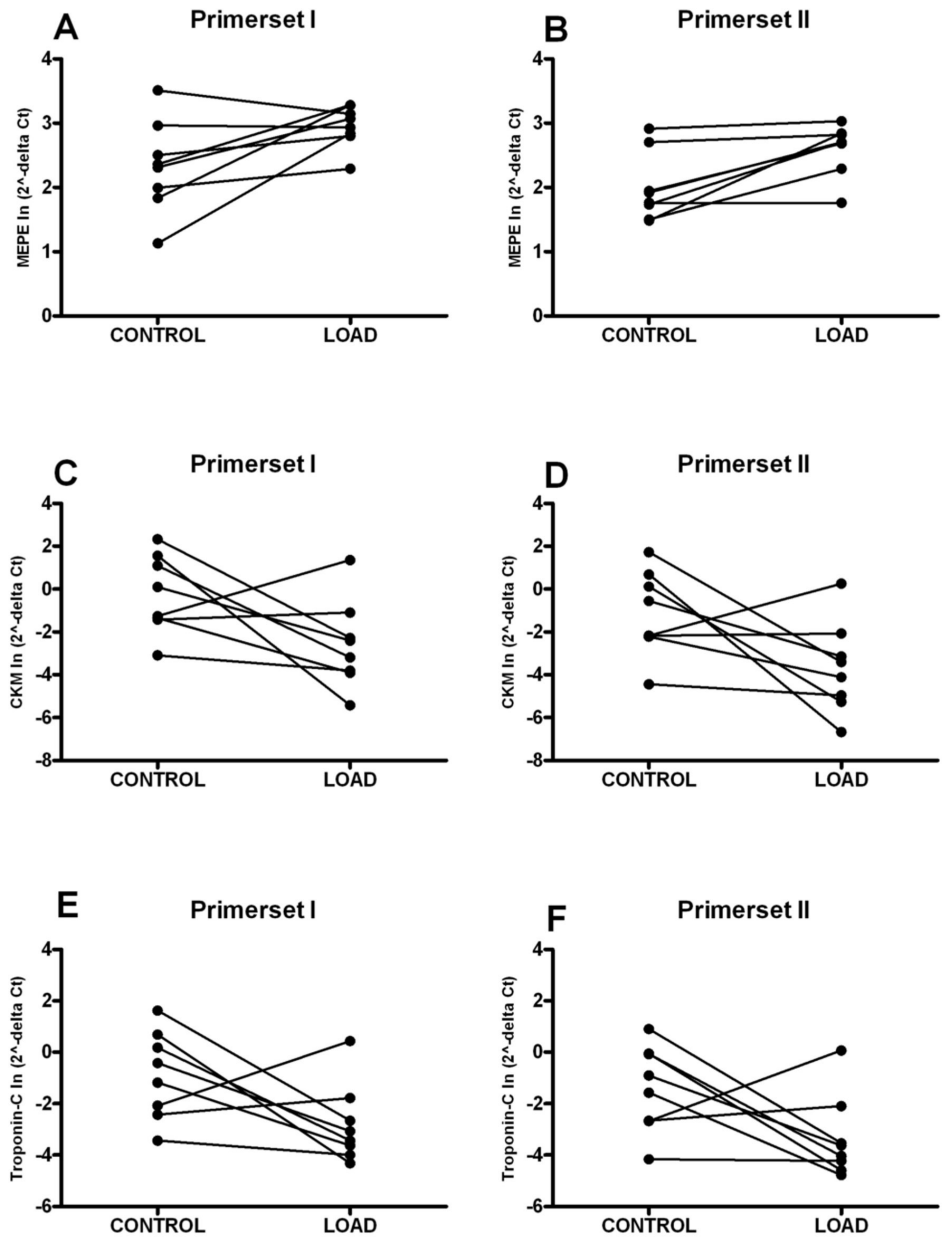
Real-time RT-PCR results of MEPE (A‑B), creatinine kinase (muscle form) (C‑D) and troponin‑C (E‑F) mRNA expression in the loaded tibiae and their contralateral controls represented per rat. Different markers represent different rats. Lines connect the right and left tibia of the same rat. For each gene, two primers were used, primer set I, which contained a sequence of the oligonucleotide as spotted on the microarray (A, C, E) and primer set II, which contained a fragment of the gene that was not present on the microarray (B, D, F). ^a^ P < 0.05; ^b^ 0.05 <P < 0.10;.

Real-time RT-PCR analysis confirmed the significant up-regulation of *MEPE* (P < 0.05) and the tendency to down-regulation of *creatine kinase* (*muscle form*) and *troponin-C* (0.05 < P < 0.10) in the LOAD tibia compared to its contra lateral control tibia (n=8) using the two different primer sets per gene (Figure 1; table 4). No significant differences of *MEPE*, *creatine-kinase* (*muscle form*) and *troponin-C* were found in the SHAM tibia compared to its contra lateral control tibia (n=10)(table 4). The other genes did not show significant differences in gene-expression between the LOAD tibia and its contra lateral control tibia (n=8) or SHAM tibia and its contra lateral control tibia (n=10)(table 4). *Fibrinogen B beta chain* was undetectable in the tibia irrespective of their treatment (LOAD, SHAM, control) as measured with both primer sets.

**Table 4:** VALIDATION OF DIFFERENTIALLY EXPRESSED GENES WITH REAL-TIME RT-PCR.

		2^-δCt^(MEAN ± SD)			2^-δCt^(MEAN ± SD)			2^-δδCt^(MEAN ± SD)		
Gene name		control LOAD (n=8)	LOAD (n=8)	P-value	control SHAM (n=10)	SHAM (n=10)	P-value	SHAM (n=10)	LOAD (n=8)	P-value
MEPE	I	12.77 ± 9.65	20.02 ± 5.67	0.041^a^	16.71 ± 7.87	17.09 ± 8.27	0.909	1.15 ± 0.64	2.36 ± 1.71	0.054^b^
	II	8.43 ± 5.24	14.33 ± 4.59	0.009^a^	11.39 ± 5.90	12.13 ± 4.02	0.455	1.20 ± 0.53	2.03 ± 0.98	0.031^a^
Garnl1/Tulip 1	I	0.659 ± 0.239	0.630 ± 0.124	0.971	0.738 ± 0.259	0.787 ± 0.314	0.836	1.14 ± 0.45	1.09 ± 0.53	0.862
	II	0.060 ± 0.024	0.050 ± 0.016	0.346	0.071 ± 0.031	0.062 ± 0.033	0.431	0.97 ± 0.40	0.91 ± 0.31	0.814
Creatine kinase (muscle form)	I	2.488 ± 3.569	0.566 ± 1.355	0.067^b^	2.519 ± 4.069	1.490 ± 2.154	0.522	10.95 ± 21.38	2.00 ± 4.84	0.289
	II	1.213 ± 1.922	0.190 ± 0.446	0.062^b^	0.947 ± 1.516	0.698 ± 1.361	0.452	15.36 ± 34.94	1.65 ± 3.92	0.319
V2R2B	I	0.010 ± 0.005	0.008 ± 0.002	0.717	0.010 ± 0.004	0.010 ± 0.004	0.783	1.24 ± 0.58	1.11 ± 0.77	0.474
	II	0.019 ± 0.009	0.017 ± 0.005	0.941	0.021 ± 0.006	0.021 ± 0.006	0.892	1.03 ± 0.28	0.96 ± 0.36	0.592
Monoamine oxidase A	I	0.093 ± 0.034	0.101 ± 0.034	0.538	0.132 ± 0.040	0.127 ± 0.050	0.638	1.04 ± 0.55	1.20 ± 0.57	0.449
	II	0.144 ± 0.046	0.139 ± 0.024	0.934	0.181 ± 0.053	0.184 ± 0.076	0.778	1.03 ± 0.33	1.03 ± 0.33	0.867
Troponin-c	I	1.182 ± 1.700	0.241 ± 0.532	0.074^b^	1.105 ± 1.516	0.660 ± 1.091	0.376	6.07 ± 12.14	1.89 ± 4.30	0.398
	II	0.640 ± 0.827	0.163 ± 0.369	0.080^b^	1.353 ± 2.260	0.518 ± 0.823	0.387	9.56 ± 22.36	2.31 ± 5.42	0.481
QFG olfactory receptor	I	0.005 ± 0.003	0.005 ± 0.002	0.882	0.013 ± 0.007	0.012 ± 0.005	0.871	1.15 ± 0.72	1.12 ± 0.48	0.828
	II	0.014 ± 0.010	0.012 ± 0.006	0.586	0.043 ± 0.035	0.041 ± 0.036	0.905	1.16 ± 0.82	1.02 ± 0.55	0.759
Kinesin light chain	I	0.506 ± 0.213	0.543 ± 0.169	0.608	0.554 ± 0.147	0.563 ± 0.121	0.838	1.11 ± 0.45	1.23 ± 0.62	0.753
	II	0.595 ± 0.196	0.603 ± 0.173	0.895	0.628 ± 0.134	0.648 ± 0.155	0.732	1.06 ± 0.24	1.10 ± 0.49	0.963

I, primer set I: contained sequence of the oligonucleotide as spotted on the microarray; II, primer set II: contained a fragment of the gene which was not present on the array; δCt = Ct _GENE OF INTEREST_ – Ct _HOUSE KEEPING GENE (PGBD)_; δδCt = δCt _TREATED_ – δCt _control_;

^a^ P < 0.05; ^b^ 0.05 < P < 0.1;

 Comparison between the LOAD (n=8) and the SHAM group (n=10) showed a significant up-regulation of *MEPE* with primer set II of the 2^-δδCt^ value in the LOAD group (independent samples t-test: P < 0.05)(table 4). No differences in expression between the LOAD (n=8) and the SHAM (n=10) group were shown in the other genes (table 4).

### Single channel analysis

When comparing the contra lateral control tibia of the LOAD group with the contra lateral control tibia of the SHAM group, a false discovery rate < 20% yielded 3 significant down-regulated genes: *p21* (*c-Ki-ras*), *phosphate regulating neutral endopeptidase on the X chromosome* (*X-linked hypophosphatemia XLH*)(*PHEX*) and *amino acid transporter system A* (*ATA2*).

### Immunohistochemistry for MEPE

**Figure 2:**
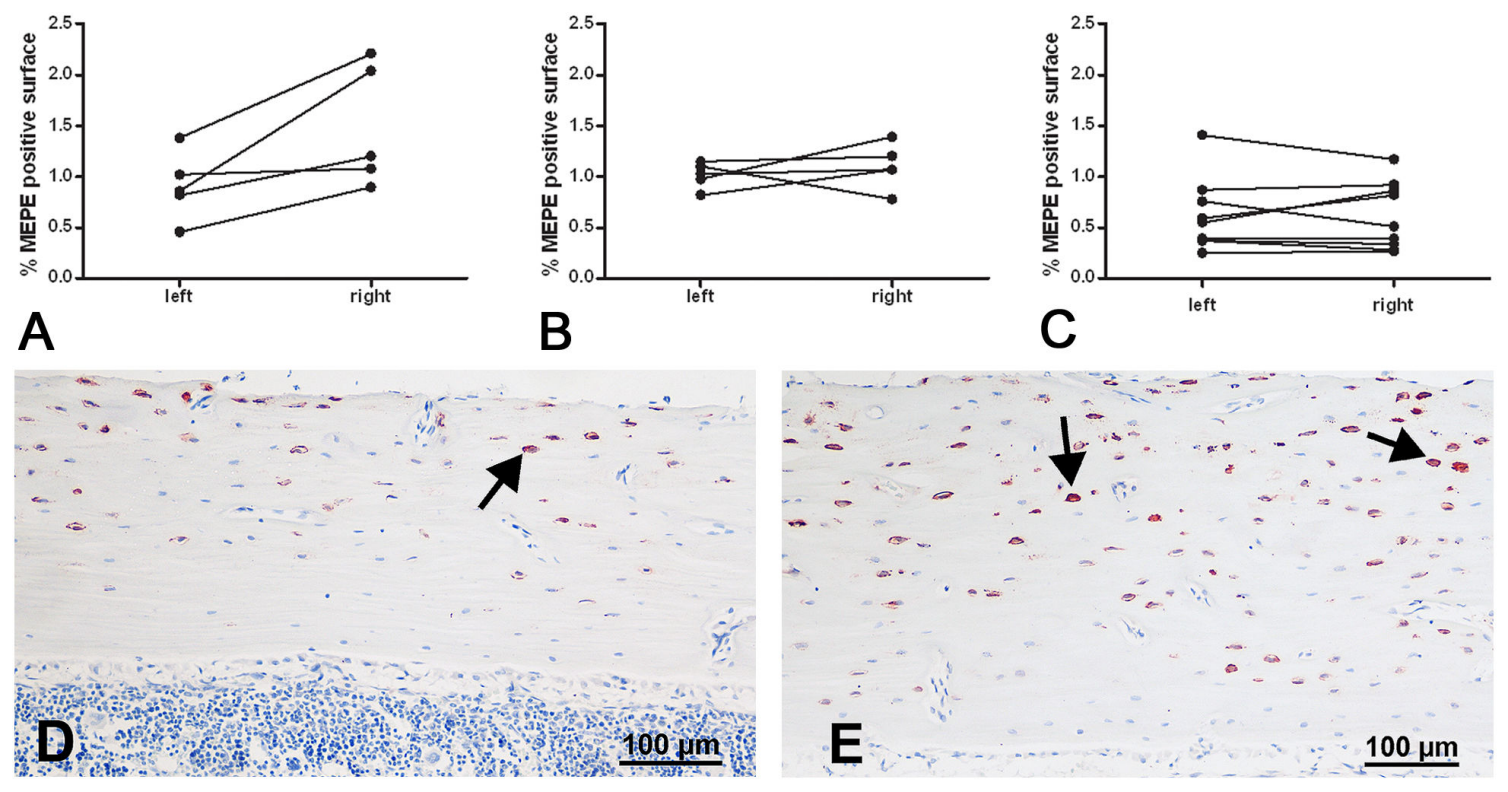
Immunohistochemistry for MEPE on sections from experiment B and C. Figures show the percentage MEPE positive area in the right and left tibiae of the individual rats after mechanical loading of only the right tibia. (A) LOAD group, (B) CONTROL group, (C) REPEATED LOAD group (D) representative MEPE staining of a left control tibia and (E) representative MEPE staining of the loaded right tibia of the same rat; arrows indicate positively stained osteocytes. bar represents 100 µm.

Protein expression of MEPE was seen primarily in the osteocytes of the cortex and the trabeculae and not in the osteoblasts on the endocortical bone surface or in the growth plate (Figure 2d). In the CONTROL group there was no significant difference in the percentage MEPE positive area between the right and left tibia (1.02 ± 0.31 vs 0.99 ± 0.12; Figure 2b). In the LOAD group the percentage MEPE positive area was higher in the tibia that had received mechanical loading compared to its contralateral left tibia as is shown in Figure 2a, the difference between the loaded tibia and the control tibia being borderline significant (1.48 ± 0.59 vs 0.92 ± 0.33; p = 0.06) After repeated mechanical loading of the right tibia for two weeks no difference in percentage MEPE positive area between the loaded tibia and the control tibia was observed (0.62 ± 0.36 vs 0.62 ± 0.33; Figure 2c). 

## Discussion

This study examined the effect of mechanical loading on the expression of multiple genes *in vivo* in rats using microarray technology. This *in vivo* study showed differential gene expression in non-pooled and non-amplificated samples of rat tibia RNA six hours after a single mechanical loading session using the 4-point-bending system. Hybridization of loaded or sham-loaded tibia versus the contra lateral control tibia of the same rat was performed. Compared to its contralateral control group, the LOAD group showed a significant up-regulation of the following genes: *MEPE*, *Garnl1*, *putative pheromone receptor V2R2B* and *isolate QFG-TN1 olfactory receptor*. Furthermore, five genes were significantly down-regulated i.e., *creatine kinase* (*muscle form*), *fibrinogen B beta polypeptide*, *monoamine oxidase A*, *troponin-C and kinesin light chain C*.

Validation with real-time RT-PCR analysis confirmed the up-regulation of *MEPE* and the down-regulation of *creatine kinase* (*muscle form*) and *troponin‑C* in the LOAD tibia compared to the contralateral control tibia six hours after a single mechanical stimulation. Because the microarrays exhibited a crossreactivity of 10%, two pairs of primers for the validation analysis were used. Both primer sets showed a similar pattern. We therefore conclude that at least three of the differentially expressed genes, *MEPE*, *creatine kinase* (*muscle form*) and *troponin-C* of the microarray analysis were specific. 

MEPE protein, as measured with immunohistochemistry, increased six hours after loading, the increase being borderline significant. This suggests that the increase in MEPE mRNA is quickly translated into protein, but it is possible that the peak in protein expression occurs at a later time point. To our surprise the level of MEPE protein in the loaded tibia was not changed after repeated loading of the tibia for two weeks, compared to the control tibia. As we did not isolate RNA from the REPEATED LOAD experiment we do not know if MEPE gene expression follows the same pattern. It is possible that the diminishing response of MEPE to mechanical loading is the result of a feedback mechanism. 

MEPE is a member of the Small Integrin Binding Ligand N-linked Glycoprotein (SIBLING) family of proteins (i.e. bone sialoprotein, osteopontin, dentin sialophosphoprotein, and dentin matrix protein)[31,32]. Members of the SIBLING family share features like the RGD motif (arginine-glycine-asparagine) and the ASARM motif (Acidic-Serine-Aspartate-Rich-MEPE)[33,34]. In rodents, MEPE mRNA and protein are expressed by osteoblasts and osteocytes [35], and in human bone by osteocytes within mineralized bone [6] and by bone-marrow cells [33]. MEPE plays a role in the regulation of bone mineralization, dentin mineralization, skeletal development, regulation of bone remodeling, renal phosphate handling and vitamin D metabolism [31,33,35]. The importance of MEPE during the bone formation after mechanical stimulation has been described earlier [27-30]. Mechanical loading induces MEPE mRNA expression in the osteocytes in a unique time-course and spatial pattern in mice [28-30]. Our results confirm these studies and show that both MEPE mRNA and protein are both increased six hours after mechanical loading. Nagel and colleagues reported that MEPE requires inducible COX‑2 to exert potent anabolic effects on normal human bone marrow osteoblast precursors [37]. It is conceivable that up-regulation of COX‑2, which is an early response to mechanical loading [8,38-41], is followed by an increased MEPE mRNA expression six hours after loading *in vivo*. Although this micro-array study did not find sclerostin to be increased after loading this has been demonstrated in literature [14,15,30]. In addition it is suggested that sclerostin is locally regulating mineralization through a MEPE-Asarm dependent mechanism [42].

The role of MEPE in bone has not yet been elucidated. MEPE-derived ASARM peptide has been shown to directly inhibit mineralization of bone tissue and also appears to inhibit sodium-dependant phosphate uptake in the kidney, thereby reducing serum phosphate concentration. On the other hand Hayashibara reported that a synthetic fragment of human MEPE (AC‑100) can stimulate new bone formation *in vivo* [36]. Overexpression of MEPE in transgenic mice resulted in decreased BMD and bone remodeling while MEPE knockout mice showed increased trabecular bone mass and increased osteoblast activity[43,44]. Whether increased MEPE acts to locally limit mineralization to prepare for increased bone formation or whether it stimulates bone formation itself needs to be further investigated. In bone tissue the actions of MEPE appear to be regulated by the presence of phosphate regulating neutral endopeptidase on the X chromosome (PHEX). This protein can bind MEPE and inhibits thereby the formation of the ASARM peptide. Interestingly, PHEX is one of the three genes that were significantly down regulated when differences between the contra lateral control tibia of the LOAD group and the contra lateral control tibia of the SHAM group were investigated in the single channel analysis. This suggests the possibility that loading of one tibia leads to simultaneous changes in the contra lateral tibia. 

Both, the microarray study and the validation with real-time RT-PCR showed that creatine kinase (muscle form) and troponin‑C were negatively regulated by mechanical stimulation. Creatine kinase (muscle form) is expressed in adult skeletal and cardiac muscle of mice [45]. It is involved in phosphocreatine biosynthesis and metabolism (Rat Genome Database). Calbindins or vitamin D dependent calcium binding proteins are members of the troponin‑C (the calcium-binding subunit of the troponin complex) superfamily [46]. Vitamin D deficiency will result in a decreased troponin‑C concentration in rabbits [47]. Our study showed that creatine kinase (muscle form) and troponin‑C are associated with the regulation of bone formation six hours after mechanical stimulation. This opens new insight into the process of bone formation after mechanical stimulation, especially for creatine kinase (muscle form) and troponin‑C.

This study showed a low number of significant differentially expressed genes at a false discovery rate of 20%. However, in microarray studies many differentially expressed genes are commonly missed when their discovery rates are slightly above the threshold [48] Our statistical analysis shows that 22 genes are differentially expressed at a false discovery rate of 50%, out of the 2375 genes that could be included in the analysis. This means that about 11 of these genes are differentially expressed and about 11 are false positives. This suggests that the number of genes that change in expression is appreciable larger than the number of genes that were discovered at a false discovery rate of 20% and confirmed by PCR. Other relevant genes besides the 2375 are not included in the analysis because they are not on the microarray or did not meet filter criteria, it is likely that many genes are involved in the response to loading of the bone. This is in agreement with the notion that many biological factors are involved in the process of bone formation (see Introduction).

Unless gene expression changes are very large, differentially expressed genes are often difficult to detect with microarrays. This is among others caused by biological variability commonly encountered in gene expression, the noise in microarray measurements which necessitates extensive filtering of the spots to maintain quality and the need to control statistically for multiple testing of the many thousands of probes. Our experimental set up is unique since we have an intra-animal control instead of a reference RNA control pool. In this model the treated right tibia is compared to the contra lateral control tibia which resulted in less noise. On the other hand the selection criteria of the spots were very severe. Of the 4803 genes, 2375 genes were left after selection. Some mechanosensitive candidate genes, as mentioned in the introduction, were lost due to filtering criteria. However the IGF-I spot was present after selection. Apparently, it is not possible with the microarray analysis to detect the 2-fold increase of IGF I as shown with in situ hybridization [18]. This 2-fold increase was restricted to a specific location in the tibia, i.e. in the osteocytes at the endosteal side of the shaft, whereas with the microarray analysis the RNA of a larger part of the shaft is used and therefore a local difference may not be detected. Xing and colleagues [49] also described the effects of 4-point-bending using the microarray technique. They used the mouse as animal model, which has the advantage of exploring 20,280 transcripts at once; however, a disadvantage of their model system is the low amount of RNA per tibia and data of single tibia were therefore not available. The experimental setup of their study was different from our study. They analyzed gene expression 24 hours after 4 days of multiple loading sessions, whereas we studied the osteogenic response six hours after a single loading session. So, the genes in their study may include genes up- or down-regulated between 24 hours and 5 days after loading, i.e. a complex cascade of genes, whereas in our study direct is restricted to the effects 6 hours after one single mechanical loading session were analyzed. Only one gene, (mono) amine oxidase A, was differentially expressed in both studies.

Since the loading consisted of bending and squeezing and the sham-loading consisted of squeezing, comparing the results of loaded tibia with the sham-loaded tibia will reveal the pure effect of bending. The real-time RT-PCR analysis showed a significant up-regulation of *MEPE* and down-regulation of *creatine kinase* (*muscle form*) and *troponin‑C* in the LOAD tibia compared to the contra lateral control tibia and did not show a significant change of expression between the SHAM tibia and the contra lateral control tibia. Thus, the increase of *MEPE* expression and decrease of *creatine kinase* (*muscle form*) and *troponin‑C* expression appears to be due to bending.

A limitation of our study is the fact that MEPE protein detection by IHC was not investigated after SHAM loading. Since comparison of the SHAM tibia and the contralateral control did not reveal any differentially expressed genes, we decided to exclude this group in the IHC experiments. Staining of capillary blood vessels was also excluded from our measurements, because osteocytes are thought to be responsible for relaying the mechanical loading signal. To detect differences in the level of MEPE protein production by osteocytes we measured MEPE positive stained bone area instead of MEPE positive osteocytes, since this is a more accurate method. 

We used the 4-point bending system which results in lamellar bone formation at the endosteal side of the shaft. Validation of the 4-point-bending system at our laboratory showed bone formation at the endosteal surface of the rat tibia 15 days after a single loading session [18] An advantage is the non-invasive application of a mechanical load to the tibia. The magnitude of the force, frequency, duration and total cycles of loading can be adjusted. However a disadvantage is the woven bone formation at the periosteal side of the shaft. An alternative for the 4-point bending model is the non-invasive ulna model, an axial compression model, in which woven bone formation at the periosteal side of the ulna is not observed.

In conclusion, the present study shows that expression of MEPE mRNA and protein, are changed during translation of mechanical stimuli six hours after mechanical loading. This could indicate that modulation of bone mineralization might play a role in the response of bone after mechanical stimulation. This study also shows that microarray experiments are helpful to identify new genes such as creatine kinase (muscle form) and troponin-C, which could have a role in bone formation after mechanical stimulation.

## References

[1] Cowin SC, Moss-Salentijn L, Moss ML (1991) Candidates for the mechanosensory system in bone. J_Biomech_Eng 113: 191-197. PubMed: 1875693. 1875693 10.1115/1.2891234

[2] Nomura S, Takano-Yamamoto T (2000) Molecular events caused by mechanical stress in bone. Matrix Biol 19: 91-96. doi:10.1016/S0945-053X(00)00050-0. PubMed: 10842092. 10842092

[3] Lanyon LE (1992) Control of bone architecture by functional load bearing. J_Bone Miner_Res 7 Suppl 2: S369-S375. PubMed: 1485545. 1485545 10.1002/jbmr.5650071403

[4] Skerry TM, Bitensky L, Chayen J, Lanyon LE (1989) Early strain-related changes in enzyme activity in osteocytes following bone loading in vivo. J_Bone Miner_Res 4: 783-788. PubMed: 2816520. 2816520 10.1002/jbmr.5650040519

[5] Lean JM, Mackay AG, Chow JW, Chambers TJ (1996) Osteocytic expression of mRNA for c-fos and IGF-I: an immediate early gene response to an osteogenic stimulus. Am_J_Physiol 270: E937-E945. PubMed: 8764176. 8764176 10.1152/ajpendo.1996.270.6.E937

[6] Raab-Cullen DM, Thiede MA, Petersen DN, Kimmel DB, Recker RR (1994) Mechanical loading stimulates rapid changes in periosteal gene expression. Calcif_Tissue Int 55: 473-478. doi:10.1007/BF00298562. PubMed: 7895187. 7895187

[7] Davidovitch Z, Shanfeld JL, Montgomery PC, Lally E, Laster L et al. (1984) Biochemical mediators of the effects of mechanical forces and electric currents on mineralized tissues. Calcif_Tissue Int 36 Suppl 1: S97 PubMed: 6204728. 10.1007/BF02406140 6204728

[8] Forwood MR (1996) Inducible cyclo-oxygenase (COX-2) mediates the induction of bone formation by mechanical loading in vivo. J_Bone Miner_Res 11: 1688-1693. PubMed: 8915776. 8915776 10.1002/jbmr.5650111112

[9] Pitsillides AA, Rawlinson SC, Suswillo RF, Bourrin S, Zaman G et al. (1995) Mechanical strain-induced NO production by bone cells: a possible role in adaptive bone (re)modeling? FASEB J 9: 1614-1622. PubMed: 8529841. 8529841 10.1096/fasebj.9.15.8529841

[10] Bravenboer N, Engelbregt MJ, Visser NA, Popp-Snijders C, Lips P (2001) The effect of exercise on systemic and bone concentrations of growth factors in rats. J_Orthop_Res 19: 945-949. PubMed: 11562145. 11562145 10.1016/S0736-0266(01)00026-2

[11] Lean JM, Jagger CJ, Chambers TJ, Chow JW (1995) Increased insulin-like growth factor I mRNA expression in rat osteocytes in response to mechanical stimulation. Am_J_Physiol 268: E318-E327. PubMed: 7864109. 7864109 10.1152/ajpendo.1995.268.2.E318

[12] Mason DJ, Suva LJ, Genever PG, Patton AJ, Steuckle S et al. (1997) Mechanically regulated expression of a neural glutamate transporter in bone: a role for excitatory amino acids as osteotropic agents? Bone 20: 199-205. doi:10.1016/S8756-3282(96)00386-9. PubMed: 9071469. 9071469

[13] Robinson JA, Chatterjee-Kishore M, Yaworsky PJ, Cullen DM, Zhao W et al. (2006) Wnt/beta-catenin signaling is a normal physiological response to mechanical loading in bone. J Biol Chem 281(42): 31720-31728. doi:10.1074/jbc.M602308200. PubMed: 16908522. 16908522

[14] Tu X, Rhee Y, Condon KW, Bivi N, Allen MR et al. (2012) Sost downregulation and local Wnt signaling are required for the osteogenic response to mechanical loading. Bone. 50(1): 209-217. doi:10.1016/j.bone.2011.10.025. PubMed: 22075208. 22075208 PMC3246572

[15] Robling AG, Niziolek PJ, Baldridge LA, Condon KW, Allen MR et al. (2008) Mechanical stimulation of bone in vivo reduces osteocyte expression of Sost/sclerostin. J Biol Chem 29;283(9): 5866-5875. PubMed: 18089564. 10.1074/jbc.M705092200 18089564

[16] Forwood MR, Bennett MB, Blowers AR, Nadorfi RL (1998) Modification of the in vivo four-point loading model for studying mechanically induced bone adaptation. Bone 23: 307-310. doi:10.1016/S8756-3282(98)00090-8. PubMed: 9737355. 9737355

[17] Turner CH, Akhter MP, Raab DM, Kimmel DB, Recker RR (1991) A noninvasive, in vivo model for studying strain adaptive bone modeling. Bone 12: 73-79. doi:10.1016/8756-3282(91)90003-2. PubMed: 2064843. 2064843

[18] Reijnders CM, Bravenboer N, Tromp AM, Blankenstein MA, Lips P (2007) Effect of mechanical loading on insulin-like growth factor-I gene expression in rat tibia. J_Endocrinol 192: 131-140. doi:10.1677/joe.1.06880. PubMed: 17210750. 17210750

[19] Reijnders CM, Bravenboer N, Holzmann PJ, Bhoelan F, Blankenstein MA et al. (2007) In vivo mechanical loading modulates insulin-like growth factor binding protein-2 gene expression in rat osteocytes. Calcif_Tissue Int 80: 137-143. doi:10.1007/s00223-006-0077-4. PubMed: 17308996. 17308996 PMC1914289

[20] Buermans HP, Redout EM, Schiel AE, Musters RJ, Zuidwijk M et al. (2005) Micro-array analysis reveals pivotal divergent mRNA expression profiles early in the development of either compensated ventricular hypertrophy or heart failure. Physiol Genomics 21: 314-323. doi:10.1152/physiolgenomics.00185.2004. PubMed: 15728335. 15728335

[21] Snijders AM, Nowak N, Segraves R, Blackwood S, Brown N et al. (2001) Assembly of microarrays for genome-wide measurement of DNA copy number. Nat_Genet 29: 263-264. doi:10.1038/ng754. PubMed: 11687795. 11687795

[22] Smyth GK, Michaud J, Scott HS (2005) Use of within-array replicate spots for assessing differential expression in microarray experiments. Bioinformatics. 21: 2067-2075. doi:10.1093/bioinformatics/bti270. PubMed: 15657102. 15657102

[23] Benjamini Y, Hochberg Y (1995) Controlling the False Discovery Rate - A Practical and Powerful Approach to Multiple Testing. J R Stat Soc B_Stat Methodol 57: 289-300.

[24] Reiner A, Yekutieli D, Benjamini Y (2003) Identifying differentially expressed genes using false discovery rate controlling procedures. Bioinformatics. 19: 368-375. doi:10.1093/bioinformatics/btf877. PubMed: 12584122. 12584122

[25] Smyth GK (2005) Paper 116: Individual channel analysis of two-colour microarrays. In 55th Session of the International Statistics Institute, 5-12 April 2005, Sydney Convention & Exhibition Centre, Sydney, Australia (CD). International Statistical Institute, Bruxelles

[26] Forwood MR, Owan I, Takano Y, Turner CH (1996) Increased bone formation in rat tibiae after a single short period of dynamic loading in vivo. Am_J_Physiol 270: E419-E423. PubMed: 8638687. 8638687 10.1152/ajpendo.1996.270.3.E419

[27] Amir LR, Jovanovic A, Perdijk FB, Toyosawa S, Everts V et al. (2007) Immunolocalization of sibling and RUNX2 proteins during vertical distraction osteogenesis in the human mandible. J_Histochem_Cytochem 55: 1095-1104. doi:10.1369/jhc.6A7162.2007. PubMed: 17625229. 17625229 PMC3957525

[28] Gluhak-Heinrich J, Yang W, Bonewald LF, Robling AG, Turner CH et al. (2005) Mechanically Induced DMP1 and MEPE Expression in Osteocytes: Correlation to Mechanical Strain, Osteogenic Response and Gene Expression Threshold. J_Bone Miner_Res 20: S73.

[29] Gluhak-Heinrich J, Pavlin D, Yang W, Macdougall M, Harris SE (2007) MEPE expression in osteocytes during orthodontic tooth movement. Arch_Oral Biol 52: 684-690. doi:10.1016/j.archoralbio.2006.12.010. PubMed: 17270144. 17270144 PMC1868431

[30] Kulkarni RN, Bakker AD, Everts V, Klein-Nulend J (2010) Inhibition of osteoclastogenesis by mechanically loaded osteocytes: involvement of MEPE. Calcif Tissue Int 87: 461-468. doi:10.1007/s00223-010-9407-7. PubMed: 20725825. 20725825 PMC2964475

[31] Fisher LW, Torchia DA, Fohr B, Young MF, Fedarko NS (2001) Flexible structures of SIBLING proteins, bone sialoprotein, and osteopontin. Biochem_Biophys_Res_Commun 280: 460-465. doi:10.1006/bbrc.2000.4146. PubMed: 11162539. 11162539

[32] Qin C, Brunn JC, Cook RG, Orkiszewski RS, Malone JP et al. (2003) Evidence for the proteolytic processing of dentin matrix protein 1. Identification and characterization of processed fragments and cleavage sites. J_Biol_Chem 278: 34700-34708. PubMed: 12813042. 12813042 10.1074/jbc.M305315200

[33] Rowe PS, de Zoysa PA, Dong R, Wang HR, White KE et al. (2000) MEPE, a new gene expressed in bone marrow and tumors causing osteomalacia. Genomics 67: 54-68. doi:10.1006/geno.2000.6235. PubMed: 10945470. 10945470

[34] Rowe PS (2004) The wrickkened pathways of FGF23, MEPE and PHEX. Crit Rev_Oral Biol_Med 15: 264-281. doi:10.1177/154411130401500503. PubMed: 15470265. 15470265 PMC3361894

[35] Petersen DN, Tkalcevic GT, Mansolf AL, Rivera-Gonzalez R, Brown TA (2000) Identification of osteoblast/osteocyte factor 45 (OF45), a bone-specific cDNA encoding an RGD-containing protein that is highly expressed in osteoblasts and osteocytes. J_Biol_Chem 275: 36172-36180. PubMed: 10967096. 10967096 10.1074/jbc.M003622200

[36] Hayashibara T, Hiraga T, Yi B, Nomizu M, Kumagai Y et al. (2004) A synthetic peptide fragment of human MEPE stimulates new bone formation in vitro and in vivo. J_Bone Miner_Res 19: 455-462. PubMed: 15040834. 15040834 10.1359/JBMR.0301263

[37] Nagel DE, Khosla S, Sanyal A, Rosen DM, Kumagai Y et al. (2004) A fragment of the hypophosphatemic factor, MEPE, requires inducible cyclooxygenase-2 to exert potent anabolic effects on normal human marrow osteoblast precursors. J_Cell Biochem 93: 1107-1114. doi:10.1002/jcb.20249. PubMed: 15449321. 15449321

[38] Bakker AD, Klein-Nulend J, Burger EH (2003) Mechanotransduction in bone cells proceeds via activation of COX-2, but not COX-1. Biochem_Biophys_Res_Commun 305: 677-683. doi:10.1016/S0006-291X(03)00831-3. PubMed: 12763047. 12763047

[39] Kawata A, Mikuni-Takagaki Y (1998) Mechanotransduction in stretched osteocytes--temporal expression of immediate early and other genes. Biochem_Biophys_Res_Commun 246: 404-408. doi:10.1006/bbrc.1998.8632. PubMed: 9610372. 9610372

[40] Ogasawara A, Arakawa T, Kaneda T, Takuma T, Sato T et al. (2001) Fluid shear stress-induced cyclooxygenase-2 expression is mediated by C/EBP beta, cAMP-response element-binding protein, and AP-1 in osteoblastic MC3T3-E1 cells. J_Biol_Chem 276: 7048-7054. PubMed: 11092885. 11092885 10.1074/jbc.M008070200

[41] Wadhwa S, Choudhary S, Voznesensky M, Epstein M, Raisz L et al. (2002) Fluid flow induces COX-2 expression in MC3T3-E1 osteoblasts via a PKA signaling pathway. Biochem_Biophys_Res_Commun 297: 46-51. doi:10.1016/S0006-291X(02)02124-1. PubMed: 12220506. 12220506

[42] Atkins GJ, Rowe PS, Lim HP, Welldon KJ, Ormsby R et al. (2011) Sclerostin is a locally acting regulator of late-osteoblast/preosteocyte differentiation and regulates mineralization through a MEPE-ASARM-dependent mechanism. J Bone Miner Res;26(7): 1425-1436. doi:10.1002/jbmr.345. PubMed: 21312267. 21312267 PMC3358926

[43] David V, Martin A, Hedge AM, Rowe PS (2009) Matrix extracellular phosphoglycoprotein (MEPE) is a new bone renal hormone and vascularization modulator. Endocrinology.150: 4012-4023. doi:10.1210/en.2009-0216. PubMed: 19520780. 19520780 PMC2819738

[44] Gowen LC, Petersen DN, Mansolf AL, Qi H, Stock JL et al. (2003) Targeted disruption of the osteoblast/osteocyte factor 45 gene (OF45) results in increased bone formation and bone mass. J Biol Chem 278: 1998-2007. doi:10.1074/jbc.M203250200. PubMed: 12421822. 12421822

[45] Zhao J, Schmieg FI, Simmons DT, Molloy GR (1994) Mouse p53 represses the rat brain creatine kinase gene but activates the rat muscle creatine kinase gene. Mol_Cell Biol 14: 8483-8492. PubMed: 7969181. 7969181 10.1128/mcb.14.12.8483-8492.1994 PMC359387

[46] Gross M, Kumar R (1990) Physiology and biochemistry of vitamin D-dependent calcium binding proteins. Am_J_Physiol 259: F195-F209. PubMed: 2201202. 2201202 10.1152/ajprenal.1990.259.2.F195

[47] Pointon JJ, Francis MJ, Smith R (1979) Effect of vitamin D deficiency on sarcoplasmic reticulum function and troponin C concentration of rabbit skeletal muscle. Clin_Sci_(Lond) 57: 257-263. PubMed: 113165. 113165 10.1042/cs0570257

[48] Taylor J, Tibshirani R, Efron B (2005) The ‘miss rate’ for the analysis of gene expression data. Biostatistics 6: 111-117 ) doi:10.1093/biostatistics/kxh021. PubMed: 15618531. 15618531

[49] Xing W, Baylink D, Kesavan C, Hu Y, Kapoor S et al. (2005) Global gene expression analysis in the bones reveals involvement of several novel genes and pathways in mediating an anabolic response of mechanical loading in mice. J_Cell Biochem 96: 1049-1060. doi:10.1002/jcb.20606. PubMed: 16149068. 16149068

